# Awareness and Knowledge of Endocrine-Disrupting Chemicals Among Pregnant Women and New Mothers: A Cross-Sectional Survey Study

**DOI:** 10.3390/toxics12120890

**Published:** 2024-12-08

**Authors:** Esin Okman, Sıddika Songül Yalçın

**Affiliations:** 1 Department of Pediatrics, Bilkent City Hospital, Ankara 06800, Türkiye; 2 Department of Social Pediatrics, Institute of Child Health, Hacettepe University, Ankara 06230, Türkiye; 3 Division of Social Pediatrics, Department of Pediatrics, Hacettepe University İhsan Doğramacı Children’s Hospital, Ankara 06230, Türkiye

**Keywords:** endocrine-disrupting chemicals (EDCs), environmental pollutants, cross-sectional survey, early-life exposure, phthalates, bisphenol A (BPA), parabens

## Abstract

Background/Objectives: Endocrine-disrupting chemicals (EDCs) are exogenous substances that interfere with hormone regulation, leading to adverse health outcomes. Despite the wide use of EDCs in daily products like plastics, personal care items, and food packaging, public awareness remains low. Pregnant women and new mothers are particularly vulnerable, as exposure to EDCs during early life stages can have long-term health impacts. This cross-sectional, questionnaire-based study aimed to assess the awareness of EDCs among pregnant women and new mothers at a maternity hospital. Methods: This cross-sectional study was conducted in a tertiary care hospital between January and August 2022. A questionnaire based on the Mutualités Libres/AIM 2020 survey was used to assess awareness of EDCs among pregnant and postpartum women. The original survey was adapted culturally and linguistically for the Turkish population through translation and expert review. The questionnaire included sections on sociodemographics, habits, knowledge, information sources, healthcare, readiness for change, expectations, and attitudes. Results: The results showed that 59.2% of participants were unfamiliar with EDCs, and many lacked awareness of the associated health risks, including cancers, infertility, and developmental disorders in children. A significant portion of respondents had never heard of bisphenol A (BPA) or phthalates, while awareness of parabens was relatively higher. Conclusions: The study concluded that increasing awareness of EDCs is essential for fostering informed avoidance behaviors, especially in vulnerable populations like pregnant women and new mothers. Public health campaigns and healthcare provider involvement are crucial for enhancing awareness and reducing the health risks associated with EDCs.

## 1. Introduction

Scientific studies regarding the adverse effects of endocrine disruptors increase every day [1,2,3,4,5,6,7]. According to the definition provided by the World Health Organization, endocrine-disrupting chemicals (EDCs) are exogenous substances or combinations that alter the functioning of the endocrine system, thus causing adverse outcomes in a healthy organism and on its subsequent generations or subpopulation [8]. These chemicals alter the production, release, binding, transport, activity, degradation, and excretion of hormones, and they impede the regular functioning of the endocrine system. Irrespective of dose, EDCs may disturb multiple systems simultaneously, even with miniscule amounts of exposure. Despite the fact that the results of early age exposures are known to be more persistent, the interval between EDC exposure and disease development varies, making it difficult to detect diseases caused by EDCs [9]. EDCs are known to be effective in various types of cancers (testicular, prostate, thyroid, breast), reproductive dysfunctions (precocious puberty, infertility), diabetes, obesity, neurodevelopmental diseases (such as autism and attention deficit hyperactivity disorder), Parkinson’s, Alzheimer’s, preterm birth, and asthma. EDCs are substances to which humans are exposed daily, making it almost impossible to completely avoid them. These chemicals are prevalent in a wide range of products, including industrial goods, agricultural products, plastics, personal care items, food packaging, flame retardants, cleaning supplies, and medical devices. Common environmental pollutants like phthalates, bisphenols, and parabens are well-documented for their endocrine-disrupting effects [10,11].

Despite the widespread presence of EDCs, public awareness regarding their harmful effects remains relatively low [6,12,13,14,15,16]. Consequently, individuals often do not adopt protective behaviors to minimize their exposure. This is particularly concerning for pregnant women, who are at risk of exposure to environmental pollutants such as pesticides, phthalates, bisphenol A, perfluorinated chemicals, heavy metals, and air contaminants [6,17,18]. The vulnerability is heightened during pregnancy and early life stages, as exposure to EDCs during critical periods of development can result in long-term, detrimental health effects on fetuses and infants.

Given the growing concerns around EDC exposure during pregnancy and early childhood, it is crucial to assess the level of awareness and knowledge among those most affected. We hypothesize that both pregnant women and new mothers have limited awareness of the risks associated with EDCs, which may hinder the adoption of protective measures. This study aims to determine the level of awareness and knowledge of EDCs among pregnant women and new mothers. By identifying knowledge gaps, the study can contribute to the development of more effective public health interventions aimed at minimizing EDC exposure during pregnancy and early childhood, ultimately promoting better long-term health outcomes for mothers and their children.

## 2. Materials and Methods

### 2.1. Study Design and the Construction of the Questionnaire

This study was conducted in a tertiary care maternity hospital, between January and August 2022, after obtaining approval from Ethics Committee of the Ankara Bilkent City Hospital (El-21-1134; 08 December 2021).

This study was designed as a descriptive, questionnaire-based survey, primarily utilizing 5-point and 3-point Likert scale questions to assess awareness of EDCs. The questionnaire consisted of 24 questions, adapted from the survey developed by Mutualités Libres and the International Association of Mutual Benefit Societies (AIM, Brussels, Belgium), which was originally used to measure the awareness of EDCs among Belgian citizens in 2020 [19].

The survey questions were restructured to consider sociocultural factors during the translation process into Turkish. Two independent translators conducted the translation, and their versions were compared for consistency to produce a consolidated translation. This final version was then back-translated into English by a third individual and compared with the original to ensure accuracy. The qualifications of all translators, with respect to national and international language examinations, were documented. Once the questionnaire was prepared, it was reviewed by environmental pollutant experts holding PhD degrees in Social Pediatrics. These experts ensured the relevance of the questions to the topic and their sociocultural appropriateness for the study participants.

### 2.2. Sample Size

To determine the required sample size for detecting a frequency of awareness in a group with an alpha of 0.05 and a power of 95%, with a proportion of 0.5 (maximum variability) and an effect size of 0.1 in a two-tailed analysis (proportion: difference from constant, a binomial test), a total of 327 cases were necessary (G*power 3.1.9.4, Franz Faul, Universität Kiel, Germany). Considering a 15% expected survey non-response rate, we planned to collect 380 completed surveys.

### 2.3. Study Group

The participants were chosen from puerperant or pregnant women aged between 18 and 49 years. The mothers were interviewed within the first week following delivery and pregnant women were interviewed when they were hospitalized for any health problems at any gestational age. Those whose native language was not Turkish were excluded from the study. Informed consent of the participants was obtained before conducting the questionnaire.

Before starting the questionnaire, the content was outlined and a brief explanation of what it was intended to investigate was given without excessive detail; the word ‘endocrine disruptors’ was not explicitly referenced. This approach was adopted to avoid participants already answering in a certain way in the ‘habits survey’ section.

### 2.4. Structure of the Questionnaire

The questionnaire was comprised of eight sections exploring; sociodemographic characteristics, habits [Question 4 (Q4); one question, 12 items], knowledge (8 questions), information sources (3 questions), healthcare (2 questions), readiness for change (one question for 12 items of Q4), expectations (5 questions), and changed attitude of the participants after completing the questions (1 question). Questions by section are referenced in Table S1. In the section on habits, we examined the frequency of habits of the participants that may pose a risk with regard to endocrine disruptors [19]. In the knowledge section, the scope was to examine the general level of knowledge concerning endocrine disruptors. Participants’ habits were assessed using the question ‘Q4. How often have you performed the following actions in the past few months?’ Subsequently, awareness of endocrine disruptors was evaluated with the question ‘Q5. Have you already heard about endocrine disruptors?’ This formed the first dependent variable of the study. After Question 5, participants were provided with brief information: ‘If you haven’t heard of them, endocrine disruptors are chemical substances found in many products. They mimic hormones in the body and may cause health problems.’ Following this explanation, participants’ sensitivity to the topic was assessed using the question ‘Q7. To what extent are you aware of the following health effects of endocrine disruptors?’ This question constituted the second dependent variable [19]. In the information sources section, we aimed to identify the sources of information used by the participants. In the healthcare survey section, we questioned whether the participants recognized the issue as an important health problem. The objective of the readiness for change section was to determine the extent of the participants’ willingness to make personal effort to change the habits that are possibly harmful for themselves. In the section on expectations, the participants were asked about their expectations from the government, non-governmental organizations, and health professionals (Table S1).

### 2.5. Statistical Analysis

The responses to the questions were presented as frequencies (n) and percentages. When evaluating the answers to the questions, risk scores were determined by assigning points to the choices of some questions. In the section where habits were evaluated, the answers to question 4 as ‘Always’ and ‘Often’ were evaluated together as a ‘Yes’ and received higher scores while the other responses were accepted as a ‘No’ and received lower scores; those with the highest scores according to the choices had lower risk. In question 5 the answers ‘Yes, I have heard of it and I know what it is.’ and ‘Yes, I have heard of it but I do not know exactly what it is.’ were adopted as a ‘Yes’. In question 7 the answers ‘fully aware’ and ‘a little aware’ were considered as a ‘Yes’ and a risk point was assigned. Those with the lowest risk score were considered to be aware of the issue.

The relationships between participants’ risky behaviors and the questions ‘Having heard about EDCs’ and ‘Awareness of the health impacts of EDCs’ were analyzed using the chi-square test. For examining differences in the relationship between their knowledge of bisphenol A, phthalates, and parabens and their awareness of the health effects of EDCs (a three-group comparison), residual analysis with Bonferroni correction was performed.

The data were analyzed using the IBM-SPSS for Windows 23.0 (SPSS Inc., Chicago, IL, USA), with a significance level of *p* < 0.05.

## 3. Results

Of the 382 mothers who participated in the survey, incomplete forms were excluded. Overall, 348 individuals responded completely to all questions and were included in the study.

When sociodemographic characteristics were analyzed, 13 (3.8%) of the participants were 20 years of age or younger, 213 (61.7%) were between 21 and 30 years of age, 107 (31.0%) were between 31 and 40 years of age, and 12 (3.5%) were between 41 and 50 years of age. Regarding the number of children, 46.3% had one child, 41.4% had two children, 6.3% had three children, 4.3% had four children, and 1.4% were pregnant. As for the educational level of the participants, 6.9% were primary school graduates, 18.7% were secondary school graduates, 34.8% were high school graduates, 36.0% were university graduates, 2.3% were master’s/doctoral degree graduates, and 1.2% had received no formal education (Table 1).

While 40.8% of the participants had prior knowledge of the term “endocrine disruptors”, 62.3% indicated awareness of the potential health implications of endocrine disruptors, which the survey questioned after the brief information given after question 5 (*‘If you haven’t heard of them, endocrine disruptors are chemical substances found in many products that mimic hormones in the body and may cause health problems.’*).

Of the participants who had a university graduate/postgraduate degree, 43.3% had prior knowledge of EDCs, while this rate was 35.5% for participants who had high school degree. Among the participants who had obtained a university or postgraduate degree, awareness of the health effects of EDCs was 40.3%, and this rate was 35.6% among high school graduates. These findings indicate that the level of education of the participants did not have a significant impact on their knowledge and awareness of EDCs.

Table 2 shows the comparison between the habits of the participants that may be risky for EDCs and their status of hearing about EDCs and awareness of their health effects.

Respondents who had heard of EDCs stated that they check the composition of cosmetics before buying them, they peel non-organic fruit and vegetables before using them, and they buy toys to be made of natural materials more often than the ones who have not heard of EDCs before (*p* = 0.002, 0.030, and 0.014, respectively, Table 2).

Respondents who were aware of the health impacts of the EDCs stated that they were using biological/ecological cleaning products, checking the composition of cosmetics before buying them, and buying organic fruit and vegetables more often than the ones who were not aware of the health impacts of EDCs (*p* = 0.011, 0.000, and 0.009, respectively). Unexpectedly, even if respondents were aware of the health effects of EDCs, they were buying clothes with labels such as ‘antibacterial’ or ‘do not iron’ more often than the others (*p* = 0.026) (Table 2).

When we specifically asked about the three specific EDCs which are most commonly known in general, the majority of respondents had never heard of bisphenol A (BPA) and phthalates, while the rate of not knowing parabens was relatively lower (Table 3). Even among the respondents who had heard of EDCs, 55.6% had never heard of BPA and 64.1% had never heard of phthalates. Likewise, 57.6% of respondents who said they were aware of the health impacts of EDCs had never heard of BPA and 65.4% had never heard of phthalates (Table 3). In contrast, 10.6% of respondents had no knowledge of parabens even if they had heard of EDCs, and 16.1% of those who were aware of the potential health effects associated with EDCs had no awareness of the existence of parabens (Table 3).

Participants who had heard of EDCs and were aware of their negative health effects were significantly more likely to know about the presence of endocrine disruptors in the ingredients of the products mentioned in Table 4. Exceptionally, they were only unaware of the presence of EDCs in house dust (Table 4).

Overall, one fourth of the participants had previously searched for information about EDCs. Among the participants who had heard of EDCs, 39.6% had previously searched for information about EDCs, which was significantly higher than those who had not (*p* < 0.001). From the participants who were aware of the adverse health effects of EDCs, 33.6% of them had previously searched for information about EDCs, which was significantly higher than those who were not aware (*p* < 0.001).

**Figure 1:**
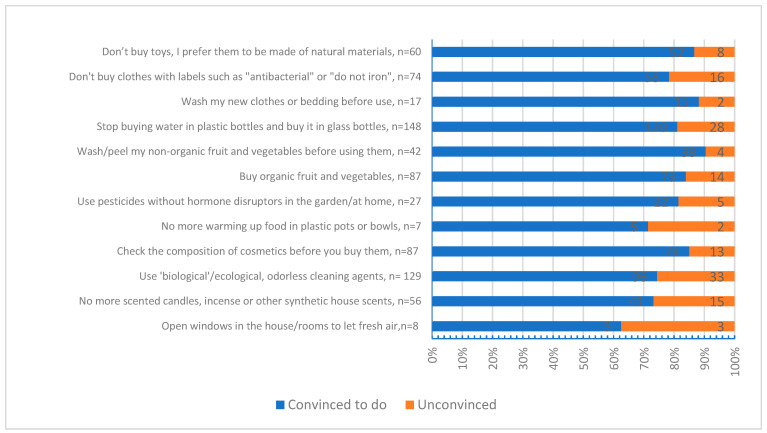
The willingness of participants with risky behaviors to change their habits; the numbers displayed in the bars represent the *n* (count) values for each category.

Figure 1 compares whether the participants who showed risky behavior in the questions about habits were convinced to change their habits, which is evaluated by the questions related to the ability to show behavioral change after being informed about endocrine disruptors during this survey. It was observed that the participants decided to change their bad habits to a great extent (Figure 1).

The respondents expressed strong expectations for government action regarding EDCs. A significant majority, 70.4%, believed that the government should launch information and awareness campaigns to educate the public on the risks of endocrine disruptors. Additionally, 64.1% felt that a ban on dangerous chemical substances was necessary. Over half of the participants, 54.0%, emphasized the importance of providing education for healthcare providers to ensure that they are well-informed and capable of sharing accurate information with the public. There was also support for government investment in research, with 43.7% advocating for funding on alternative substances and the health impacts of endocrine disruptors. Furthermore, 41.4% called for the government to support international initiatives aimed at eliminating the use of these harmful chemicals, and 38.2% believed that a national action plan to reduce the use of toxic substances in products should be developed.

Participants expressed various expectations from non-governmental organizations (NGOs) regarding information on endocrine disruptors. The majority (73.3%) expected general information about endocrine disruptors in products, while 61.9% sought awareness-raising campaigns specifically targeting vulnerable groups, such as pregnant women and young children. Nearly half (48.4%) desired concrete recommendations to help reduce exposure to endocrine disruptors in daily life, and 44.2% expected NGOs to provide accessible resources, such as brochures, mobile apps, or websites. Additionally, 38.1% preferred personalized information tailored to their specific circumstances, such as being pregnant or having young children. Notably, 7.8% of respondents reported having no expectations for information from NGOs.

When it comes to addressing information needs about EDCs, expectations vary based on prior awareness. Participants who had previously heard of EDCs were more likely to prefer concise solutions, such as product labels indicating safety, government websites, or pictograms highlighting the presence of specific chemicals in products. In contrast, those aware of the negative health effects of EDCs tended to favor more detailed and comprehensive information. These preferences included smartphone apps offering basic information about EDCs, apps tailored to their personal circumstances (e.g., pregnancy or having young children), and brochures or websites with personalized content (Table 5).

When asked whether they would prefer to receive information about endocrine disruptors from healthcare professionals, 57.8% of participants indicated that they would welcome such information, while 29.4% stated that it could be beneficial, and 12.8% expressed no desire for it. Overall, prior knowledge of EDCs or awareness of their health effects did not significantly influence participants’ expectations of healthcare professionals. However, participants who were already familiar with EDCs were significantly more likely to seek advice on minimizing exposure during pregnancy compared to those who were not (*p* = 0.020) (Table 5).

After completing the questionnaire, 53.8% of participants stated that they would actively seek more information about endocrine disruptors, 39.8% indicated that they might seek additional information, and 6.4% stated they would not. There was no statistically significant relationship between the intention to seek more information and prior awareness of EDCs or their health effects.

## 4. Discussion

This study demonstrated low levels of awareness of EDCs among the mothers who had recently given birth and pregnant women.

Habits: In daily life, we engage in activities that may lead to exposure to endocrine disruptors continuously throughout the day. This study indicates that many participants already practice certain routines that help minimize this exposure. The highest-rated beneficial habits were ventilating the house, avoiding heating food in plastic containers in the microwave, avoiding the use of pesticides/insecticides (indoor and outdoor herbicides) in the garden/the house, and washing newly purchased clothes or bedding before use. The first habit is in common with the Belgium survey [19], but in that study, the most frequently observed negative behaviors were the practice of heating food in plastic containers using a microwave and the use of newly purchased clothing without prior washing. We believe that the reasons for these discrepancies are that microwaves are not yet widely used in Türkiye, and washing newly purchased clothes and bedding is seen as an indication of meticulous attention to hygiene in Turkish culture.

In studies about the perception of ambient air pollution, the perceived adverse health effects of the household air pollution by participants are bad health conditions such as eye irritation and respiratory problems like coughing, sneezing, and asthma. Endocrine disruptive effects of house dust are not mentioned by the participants of these studies [20,21,22,23]. Likewise, our study showed the answer to the question on admitting the house dust as a source of EDCs was a ‘no’ by 85% of them. Many known and suspected EDCs are present in significant levels in indoor dust alongside various other harmful substances, potentially making residential indoor exposure a more significant source of overall exposure than diet, particularly for children [24,25,26].

Knowledge: In line with findings from the existing literature, the results of our study once again demonstrated that participants had a low level of knowledge about EDCs [12,19,27,28,29]. In our study group, 59.2% had no familiarity with this concept of endocrine disruptors and 37.7% reported a lack of awareness for potential causes of major health issues, such as certain types of cancer, infertility, obesity, and developmental problems in babies, as well as having particularly harmful effects on pregnant women and negative impacts on the growth of children and adolescents. Similarly, in a survey on endocrine disruptors conducted by the Independent Health Insurance Funds in Belgium in 2020 with the participation of 1000 individuals, 52% of the respondents had never heard of endocrine disruptors, and awareness of their health effects was present in only at a quarter of the group [19]. Similarly, the proportion of individuals who had never heard of EDCs was 54.3% in a French study [27]. In a study from Türkiye examining the environmental risk perceptions of mothers with open-ended questions, 30.2% stated that they did not know at all what the environmental risk factor was, which was the largest proportion of the participants [13]. Our results did not indicate a relation between the participants’ educational levels and knowledge about EDCs. Previous studies indicated that educated women tend to have better access to environmental health information and the resources to make informed lifestyle choices [13,27,30]. The difference might be due to differences in mother’s education level differences; 38.3% of the participants in our study had attained a university degree or higher, whereas 77% of the participants in the French study had done so [27].

Compared to their counterparts, the participants who had prior knowledge of EDCs and those who were aware of their health effects were significantly in the habit of checking the composition of cosmetics (make-up, creams, shampoo, deodorant, shaving foam, hand cream) before buying them. Considering our participants were pregnant women and new mothers, this result was promising.

Among participants who reported being aware of the negative health effects of endocrine disruptors, the statistically higher rate of responding ‘yes’ to the question regarding the purchase of clothing labeled as ‘antibacterial’ or ‘do not iron’ may be influenced by the perception that antibacterial products were more desirable during the survey period, when the most rigorous precautions against the transmission of the SARS-CoV-2 virus were in place. In a study examining parents’ concerns about environmental chemical exposure, researchers found higher urinary triclosan levels in children of parents who were more concerned about environmental chemicals, which was unexpected. The authors suggested that these parents may have chosen antimicrobial products, like hand sanitizers and cleaners, due to concerns about household microbes [31]. Even when individuals are concerned about environmental pollutants, their fear of infectious agents may override these concerns.

It is shown that phthalates in personal care products and BPA in food packaging have been linked to increased urinary concentrations, with studies showing higher levels following the use of these products and consumption of packaged foods [32,33,34]. In our study group, 65.8% of participants indicated that they were unaware of the term ‘BPA’, and 73.6% was unaware of ‘phthalates’. BPA and phthalates are relatively unknown, while parabens are somewhat more recognized. Overall, 22.1% stated that they were unaware of ‘parabens’. Even among those who claimed to have heard of EDCs or who were aware of their health impacts, the majority remained unfamiliar with phthalates, BPA, and parabens. Nearly half claimed that they check the labels of personal care products. Consequently, even if these individuals read product labels, they do not know what to look for. In the context of Türkiye, the slightly higher recognition of parabens may be attributed to the recent proliferation of “paraben-free” labels on certain medications and cosmetic products, as well as the media, social media content, blogs, and programs discussing the issue. Similarly, we are aware that BPA-free labels are becoming increasingly common, particularly on baby products like bottles and pacifiers, and on school-aged children’s items like water bottles. However, such labels for phthalates are virtually nonexistent. As a result, consumers are unable to search for phthalates, one of the most prevalent EDCs, while reading labels, making it nearly impossible to avoid them. Despite a general awareness of EDCs and their presence in various products, the ability to make healthier choices on a product-specific basis is significantly reduced when consumers are not familiar with specific names like phthalates, BPA, or parabens. Similarly, 55% of respondents in Belgium indicated that they had no prior awareness of bisphenol A, 64% of phthalates, and 28% of parabens [19]. In the study by Rouillon et al. assessing 137 pregnant women, 25.6% demonstrated awareness of BPA, while 24.1% reported awareness of parabens [27]. The question regarding label reading is open to multiple interpretations. If an individual professes to read product labels despite lacking awareness of EDCs, it is possible that they are doing so to ascertain information such as the product’s expiration date, rather than to investigate the ingredients. Verifying the expiration date is not equivalent to scrutinizing the ingredient list. In this context, the primary measures that need to be taken in Türkiye should begin with enhancing health literacy, particularly to improve label reading and the ability to identify what to look for. Increasing public awareness of what to avoid when reading labels is essential for mitigating the risks associated with exposure to harmful substances.

Sources of information and expectations: In line with the Belgian study (21%) [19], 25.8% of our participants had looked up information about EDCs. According to Eurobarometer 501, the main sources of information on environment in Europe are television news (66%), the internet (websites, blogs, forums etc.) (38%), the radio (23%), and films and documentaries (23%) [35]. Our study participants were expecting solutions to meet the information needs about EDCs; those who had heard of EDCs before were more likely to expect solutions that include more concise information, such as a label showing which product is safe, a government website, or pictograms showing that the product contains certain chemicals, while participants who were aware of the negative effects of EDCs on health were more likely to expect getting access to longer information, such as an app on their smartphone with basic information about endocrine disruptors, an app on their smartphone with information adapted to their personal situation (e.g., pregnancy, young children), or brochures/website with information adapted to their personal situation. This indicates that individuals who are aware about the health impacts of EDCs require access to more comprehensive and detailed information sources than those who had only heard of EDCs.

The most frequently expressed expectations from the government among participants were that the government should implement information and awareness campaigns to educate the public about the risks associated with endocrine disruptors and prohibit the use of harmful chemicals. The data reveal that 72% of the European population and 74% of United Kingdom population believes that national governments are failing to adequately address environmental protection [35]. Our study found that more than half of the participants expressed a desire for non-governmental organizations to provide general information on products containing endocrine disruptors, as well as to conduct information and awareness-raising campaigns, particularly targeting vulnerable groups such as pregnant women and young children.

We observed that three out of four participants expect healthcare providers (e.g., doctors, pharmacists) to provide information about endocrine disruptors. Mostly, prior knowledge of EDCs or awareness of the health effects of EDCs did not make a significant difference in expectations of health professionals, except that the participants who knew about EDCs were more likely to seek advice on endocrine disruptors to minimize exposure during pregnancy than those who did not. Unfortunately, studies investigating the knowledge and attitudes of healthcare professionals regarding environmental pollutants or endocrine disruptors have consistently found that the percentage of healthcare workers who feel adequately informed is quite low [36,37].

Willingness to change: We evaluated the effect of having prior knowledge about EDCs or awareness of their health effects on being open to change with the questions that investigated whether the participants who engaged in more risky behaviors according to their answers to the questions about habits were willing to change after the short information given in the questionnaire. Our findings indicate no significant differences between the groups. The presence or absence of prior knowledge of EDCs and awareness of their health effects did not influence the participants’ readiness to alter their risky behavior. In both groups, more than half of the participants stated that they were certainly willing to change their risky habit even if it requires time, effort, or financial resources. Although numerous studies on endocrine disruptors suggest that exposure can be minimized through personal measures, pregnant women and new mothers may lack the social and economic resources to implement these strategies under certain circumstances. Several studies also indicates that this limitation can lead to increased stress and feelings of helplessness among mothers [38,39,40,41]. Barrett et al.’s study highlights that even during pregnancy, many women do not actively reduce their exposure to environmental chemicals, either due to a lack of awareness about its importance or uncertainty on how to minimize such exposure [30].

Limitations: One of the primary limitations of our study is that the survey was conducted in a hospital setting. As a result, the sample may not be representative of the general population, which limits the generalizability of the findings to broader societal contexts. Additionally, our collection of sociodemographic data was restricted to only a few variables, including age, educational level, and the number of children. Furthermore, as with most survey-based research, there is a possibility of response bias, where participants may have provided socially desirable answers or felt compelled to respond to questions on topics they were unfamiliar with, thus potentially introducing bias into the study results. Another limitation of this study is that maternal mental health was not assessed, which may have influenced the responses, particularly as it can impact decision-making and behavior during both the postpartum period and pregnancy.

Strengths: A key strength of this study is its focus on a particularly vulnerable population—pregnant women and new mothers—who are among those most at risk from the harmful effects of EDCs. Conducting the research in a maternity hospital allowed for direct engagement with this high-risk group, ensuring relevant and targeted insights. Another notable strength is the socioeconomic diversity of the participants, which enhances the generalizability of the findings across women from various backgrounds. This diversity provides valuable insights into how socioeconomic factors influence awareness and avoidance behaviors related to EDC exposure. It also highlights the need for future public health interventions to be tailored to address the specific needs of women from different socioeconomic groups.

## 5. Conclusions

Our study highlights a generally low level of knowledge and awareness about EDCs among pregnant women and new mothers, consistent with findings in the existing literature. Although participants were already engaging in some avoidance behaviors, these actions appeared to be more instinctive than informed. This underscores the potential for improving these behaviors by increasing awareness and providing clear, accessible information about the risks associated with EDC exposure. Such efforts could make avoidance behaviors more intentional and effective, ultimately reducing exposure during critical developmental periods and promoting the health of future generations. The low levels of awareness identified in this study point to a pressing need for targeted public health campaigns and educational programs, particularly aimed at pregnant women and new mothers. Future research should prioritize developing and evaluating interventions that educate this population about EDCs and their health risks. Additionally, longitudinal studies could explore the long-term effectiveness of these interventions on avoidance behaviors and health outcomes for both mothers and their children. Expanding future research to include a more diverse sample and examining a wider range of sociodemographic factors would provide a deeper understanding of how awareness and behaviors related to EDCs vary across different social groups, offering valuable insights for tailoring public health initiatives.

## Figures and Tables

**Table 1:** Sociodemographic data of the participants.

	*n*	%
**Age (years)**		
<20	13	3.8
21–30	213	61.7
31–40	107	31.0
41–50	12	3.5
**Parity**		
Nulliparous	5	1.4
One child	161	46.3
Two children	144	41.4
Three children	22	6.3
Four children	15	4.3
**Educational level**		
No formal education	4	1.2
Primary school	24	6.9
Secondary school	65	18.7
High school	121	34.8
University	125	36.0
Master’s/doctoral degree	8	2.3
**Marital status**		
Married/cohabiting/civil partnership	348	100.0

**Table 2:** Ratio of participants’ risky behaviors according to having heard about endocrine-disrupting chemicals and having awareness about their health effects.

		Overall, % *	Having Heard About EDCs, % *			Awareness of the Health Impacts of EDCs, % *		
	Items		Yes	No	*p*	Aware	Not Aware	*p*
		*n* = 348	*n* = 142	*n* = 206		*n* = 217	*n* = 131	
P	How often do you open the windows in the house/rooms to let fresh air	93.4	95.8	91.7	0.137	95.4	90.1	0.053
N	I use scented candles, incense or synthetic (non-natural) house scents	61.2	64.8	41.3	0.255	62.2	59.5	0.620
P	I use biological/ecological cleaning products	35.1	39.4	32.0	0.155	40.1	26.7	**0.011**
P	I check the composition of cosmetics (make-up, creams, shampoo, deodorant, shaving foam, hand cream) before buying them	49.7	59.9	42.7	**0.002**	57.6	36.6	**<0.001**
N	I warm up food in plastic jars or bowls	95.4	96.5	94.7	0.426	94.9	96.2	0.589
N	I use non-natural pesticides/insecticides (indoor and outdoor herbicides) in the garden/the house	87.1	85.9	87.9	0.594	12.9	13.0	0.984
P	I buy organic fruit and vegetables	55.5	57.7	53.9	0.476	60.8	46.6	**0.009**
P	I peel my non-organic fruit and vegetables before using them	60.1	66.9	55.3	**0.030**	59.9	60.3	0.942
N	I buy water in plastic bottles	21.3	21.1	21.4	0.958	19.4	24.4	0.263
N	I use my new clothes or bedding immediately after buying them, without washing them first	76.4	81.0	73.3	0.097	78.3	73.4	0.281
P	I buy toys, I prefer them to be made of natural materials (and thus not of plastic)	44.3	52.1	38.8	**0.014**	47.9	38.2	0.076
N	I buy clothes with labels such as ‘antibacterial’ or ‘do not iron’	61.2	55.6	65.0	0.076	56.7	68.7	**0.026**

* Column percentage for each item. P = positive, N = negative (referring to the structure of the question. Positive and negative phrases were used to indicate the structure of the question. Positive answers to positive questions received higher scores, while negative answers to negative questions received higher scores. Thus, participants with higher total scores were found to be at lower risk. The percentages of those who responded negatively to negative questions and those who responded positively to positive questions are indicated in overall column). Answers to the question on ‘Opening windows for fresh air’ as ‘A few times a day’ and ‘Once a day’ were evaluated as a ‘Yes’. ‘Never’, ‘Less than once a week’, and ‘A few times a week’ were evaluated as a ‘No’. In the rest of the questions; ‘Always’ and ‘Often’ were evaluated together as a ‘Yes’, and ‘Rarely’, ‘Hardly ever’, and ‘Never’ were accepted as a ‘No’. Significant *p* values are shown in bold font.

**Table 3:** Association of having heard of EDCs and awareness of their health effects with knowing what bisphenol A, phthalates, and parabens are.

	Overall		Having Heard About EDCs, %		Awareness of the Health Impacts of EDCs, %	
Items	*n*	% ^#^	% ^&^	*p*	% ^&^	*p*
**Overall**	348	100	40.8		62.3	
**Bisphenol A**						
I know what it is and what products it can be present in	47	13.5	49.8 ^a^	**0.001**	83.0 ^a^	**<0.001**
I have heard of it, but I do not know what it means	72	20.7	55.6 ^a^		73.6 ^a^	
I do not know it	229	65.8	34.5 ^b^		54.6 ^b^	
**Phthalate**						
I know what it is and what products it can be present in	29	8.3	55.2 ^a^	**0.004**	89.7 ^a^	**<0.001**
I have heard of it, but I do not know what it means	63	18.1	55.6 ^a^		77.8 ^a^	
I do not know it	256	73.6	35.5 ^b^		55.5 ^b^	
**Paraben**						
I know what it is and what products it can be present in	184	52.9	44.0 ^a^	**0.003**	69.6 ^a^	**<0.001**
I have heard of it, but I do not know what it means	87	25.0	52.9 ^a^		62.1 ^a^	
I do not know it	77	22.1	19.5 ^b^		45.5 ^b^	

^#^ column percentage for each item; ^&^ row percentage; ^a,b^ values having different letters in the same column for the same parameter were found to be significant, *p* < 0.05. Significant *p* values are shown in bold font.

**Table 4:** Association of having heard of EDCs and awareness of their health effects with knowing the existence of EDCs in the products.

		Having Heard About EDCs, % *			Awareness of the Health Impacts of EDCs, % *		
Those who have heard about the existence of EDCs in the products mentioned below	Overall % *	Yes	No	*p*	Aware	Not Aware	*p*
***n***	348	142	206		217	131	
Non-organic fruit and vegetables	40.5	50.7	33.5	**0.001**	52.1	71.8	**<0.001**
Plastic packaging (e.g., plastic packaging material in which foodstuffs such as meat, cheese, vegetables are packed)	47.4	58.5	39.8	**0.001**	59.4	27.5	**<0.001**
Kitchen utensils: plastic pots, non-stick pans	41.7	52.8	34.0	**<0.001**	50.2	27.5	**<0.001**
Products for personal hygiene (shaving foam, make-up, hand cream)	28.4	38.7	21.4	**<0.001**	32.7	21.4	**0.023**
Textiles (clothing, bedding, etc.)	20.7	29.6	14.6	**0.001**	25.8	12.2	**0.002**
Cleaning and household products	32.9	42.6	26.2	**0.001**	40.7	19.8	**<0.001**
Children’s toys	36.9	48.6	28.8	**<0.001**	45.2	23.1	**<0.001**
Non-natural pesticides/insecticides	32.2	45.1	23.3	**<0.001**	39.6	19.8	**<0.001**
Candles and incense	23.3	34.5	15.5	**<0.001**	30.9	10.7	**<0.001**
House dust	15.0	18.3	12.7	0.149	17.5	10.8	0.089

* Column percentage for each item. Significant *p* values are shown in bold font.

**Table 5:** Relation of the solutions to meet information needs related to EDCs and expectations from the health professionals in terms of having heard about EDCs and the awareness of the health impacts.

		Having Heard About EDCs, % *			Awareness of the Health Impacts of EDCs, % *		
Solutions and Expectations Items	Overall % * *n* = 348	Yes *n* = 142	No *n* = 206	*p*	Aware *n* = 217	Not Aware *n* = 131	*p*
**Solutions to meet information needs related to EDCs**							
Application on your smartphone with basic information	64.7	70.0	61.1	0.890	70.0	56.2	**0.009**
Application on your smartphone with information adapted to your situation	81.7	84.3	79.9	0.302	85.9	74.8	**0.010**
Application that scans the barcode of a product and analyses the composition of the product	70.9	74.1	68.7	0.277	73.0	67.4	0.275
Label indicating which product is safe	81.3	87.8	77.0	**0.012**	84.4	76.3	0.061
Brochure/online information (adapted to my personal situation)	76.6	86.4	70.1	**<0.001**	84.0	64.9	**<0.001**
Website of the government	75.0	84.3	68.6	**0.001**	77.5	71.0	0.178
Pictogram indicating that the product contains certain chemical substances (e.g., “contains BPA”)	73.5	82.9	67.0	**0.001**	76.4	68.7	0.116
**Expectations from the health professionals**							
General information about endocrine disruptors in products	69.4	73.6	66.5	0.163	69.0	70.0	0.848
Concrete information and advice, based on scientific research, on how to protect myself/my children from endocrine disruptors, e.g., how to reduce endocrine disruptors in daily life	67.6	70.7	65.5	0.312	70.0	63.8	0.241
Advice on endocrine disruptors during my pregnancy to minimize exposure	49.6	57.1	44.3	**0.020**	50.7	47.7	0.588
Nothing	8.5	6.4	9.9	0.263	6.6	11.5	0.109

* Column percentage for each solutions and expectations items. Significant *p* values are shown in bold font.

## Data Availability

Due to privacy, the data presented in this study are available on request from the corresponding author.

## Supplementary Materials

The following supporting information can be downloaded at: https://www.mdpi.com/article/10.3390/toxics12120890/s1, Table S1: Content of the questionnaire by section.
