# Supplementary material for: Awareness and Knowledge of Endocrine-Disrupting Chemicals Among Pregnant Women and New Mothers: A Cross-Sectional Survey Study
Source: Toxics. 2024 Dec 8;12(12):890. doi: 10.3390/toxics12120890 (PMC11728504; doi:10.3390/toxics12120890)
Supplement: Supplementary file 1 [file toxics-12-00890-s001.zip › toxics-3295724-supplementary.pdf]

Table S1: Content of the questionnaire by sections

**Socio-demographic characteristics**	**Q1.** Age<br><br>**Q2.** Education status<br><br>**Q3.** Number of children
**Habits**	**Q4.** How often have you performed the following actions in the past few months?<br>o How often do you open the windows in the house/rooms to let fresh air<br>o I use scented candles, incense or synthetic (non-natural) house scents<br>o I use biological/ecological cleaning products<br>o I check the composition of cosmetics (make-up, creams, shampoo, deodorant, shaving foam, hand cream…) before buying them<br>o I warm up food in plastic jars or bowls<br>o I use non-natural pesticides/insecticides (indoor and outdoor herbicides) in the garden/the house<br>o I buy organic fruit and vegetables<br>o I peel my non-organic fruit and vegetables before using them<br>o I buy water in plastic bottles<br>o I use my new clothes or bedding immediately after buying them, without washing them first<br>o I buy toys, I prefer them to be made of natural materials (and thus not of plastic)<br>o I buy clothes with labels such as 'antibacterial' or 'do not iron' (e.g. non-ironable shirts, socks with antibacterial effect...)
**Knowledge**	**Q5.** Have you already heard about endocrine disruptors?<br><br>*'If you haven't heard of them, endocrine disruptors are chemical substances found in many products that mimic hormones in the body and may cause health problems.'*<br><br>**Q6.** How well informed do you feel about the potential dangers of certain endocrine disruptors in everyday products such as clothing, food, furniture, toys, cosmetics, packaging?<br><br>**Q7.** To what extent are you aware of the following health effects of endocrine disruptors?<br>o The presence of these substances can cause health problems such as some types of cancer, fertility problems, obesity<br>o The presence of these substances is especially dangerous for pregnant women, as they can affect the baby's development. This can have long-term consequences<br>o The presence of these substances may have negative effects on the development of children and adolescents (e.g. early or late puberty...).<br><br>**Q8.** To what extent do you agree with the following statement: on the Turkish market only products that do not contain potentially dangerous chemical substances are available?<br><br>**Q9.** Have you already heard of the possible presence of endocrine disruptors in the following products? (please mark with a cross)<br>o Non-organic fruit and vegetables

	o Plastic packaging (e.g. plastic packaging material in which foodstuffs such as meat, cheese, vegetables... are packed),<br>o Kitchen utensils: plastic pots, non-stick pans...<br>o Products for personal hygiene (shaving foam, make-up, hand cream...)<br>o Textiles (clothing, bedding, etc.)<br>o Cleaning and household products<br>o Children's toys<br>o Non-natural pesticides/insecticides<br>o Candles and incense<br>o House dust<br><br>**Q10.** To what extent do you know the BPA (bisphenol A)?<br>Bisphenol A is a chemical used in the production of certain plastics and resins. It can be found in plastic bottles, food packaging, canning jars, receipts, among others.<br><br>**Q11.** To what extent do you know the Phthalate?<br>Phthalates are a group of chemicals used to make certain types of plastic flexible and pliable. Phthalates can be found in many common products such as food packaging, toys, personal care products.<br><br>**Q12.** To what extent do you know the Parabens?<br>Parabens are a group of chemical substances that can be used e.g. as preservatives, perfumes or because of their antimicrobial action. They can be found in many commonly used products such as personal care products, cleaning products, medicines, etc.
**Information**	**Q13.** Are you in the habit of reading the label of the products you buy (e.g. herbicides, creams, shampoos, foodstuffs)?<br><br>**Q14.** Have you already looked up information on endocrine disruptors?<br><br>**Q15.** Which sources of information would you consult to get more information on the presence of endocrine disruptors in your daily life?<br>o Governmental websites<br>o Internet (other than governmental websites)<br>o Social media<br>o Newspapers<br>o Radio, television<br>o Friends, family<br>o Books or scientific publications<br>o Brochures<br>o Events<br>o Doctors<br>o Pharmacists<br>o Other health professional (nurses etc.)
**Health care**	**Q16.** In general, are you concerned about exposure to endocrine disruptors present in everyday products such as toys, cosmetics?<br><br>**Q17.** Who are you worried about?<br>o Myself and my family<br>o My children in particular<br>o All citizens<br>o The environment in general

**Readiness for change**	**Q18.** Below is a list of measures you can take to reduce potential exposure. Please select the option that best suits you to reduce your exposure to endocrine chemicals.<br>o Open windows in the house/rooms to let fresh air<br>o No more scented candles, incense or other synthetic (not natural) house scents.<br>o Use 'biological'/ecological, odorless cleaning agents<br>o Check the composition of cosmetics (make-up, creams, shampoo) before you buy them<br>o No more warming up food in plastic pots or bowls<br>o Use pesticides/insecticides without hormone disruptors in the garden/at home<br>o Buy organic fruit and vegetables<br>o Wash/peel my non-organic fruit and vegetables before using them<br>o Stop buying water in plastic bottles and buy it in glass bottles (or use tap water)<br>o Wash my new clothes or bedding before use<br>o Don't buy clothes with labels such as "antibacterial" or "do not iron" (e.g. non-ironable shirts, socks with antibacterial effect)<br>o Don't buy toys, I prefer them to be made of natural materials (and thus not of plastic)
**Expectations**	**Q19.** Below you will find some statements about what the government can do about endocrine disruptors. What do you expect from the government?<br>o The government should launch information and awareness campaigns about the risks of endocrine disruptors<br>o The government needs to develop a national action plan to reduce the use of toxic substances in products<br>o The government must ban dangerous chemical substances.<br>o The government should support international initiatives to eliminate the use of endocrine disruptors.<br>o The government should support research on alternative substances and on the impact of endocrine disruptors on health<br>o The government should provide education, e.g. for health care providers, so that they are well informed and can pass on the right information<br><br>**Q20.** Not only the government but also non-governmental organizations (NGOs) can inform their members about endocrine disruptors (foundations, associations, etc.). What do you expect from NGOs on endocrine disruptors?<br>o General information on endocrine disruptors in products<br>o Concrete advice on reducing endocrine disruptors in daily life<br>o Information and awareness-raising campaigns, especially for vulnerable people (pregnant women, small children)<br>o Information sources (e.g. brochures/app/website)<br>o Personalized information, adapted to my personal situation (e.g. adapted to the fact that I am pregnant, have small children)<br>o Nothing<br><br>**Q21.** Below you will find some solutions that can meet information needs on endocrine disruptors. Please mark how suitable you would find such an application for yourself.<br>o Application on your Smartphone with basic information

	o Application on your Smartphone with information adapted to your situation<br>o Application that scans the barcode of a product and analyses the composition of the product<br>o Label indicating which product is safe<br>o Brochure/ online information (adapted to my personal situation)<br>o Website of the government<br>o Pictogram indicating that the product contains certain chemical substances (e.g. "contains BPA")<br><br>**Q22.** In your opinion, do health professionals (medical doctors, pharmacists) play a role in providing information on endocrine disrupters?<br><br>**Q23.** What do you expect from your health professional concerning endocrine disruptors?<br>o General information about endocrine disruptors in products<br>o Concrete information and advice, based on scientific research, on how to protect myself/ my children from endocrine disruptors, e.g. how to reduce endocrine disruptors in daily life<br>o Advice on endocrine disruptors during my pregnancy to minimize exposure<br>o Nothing
**Changed attitude after completion**	**Q24.** Will you seek more information on endocrine disrupters after having completed this survey?
